# In Vitro Inhibitory and Proliferative Cellular Effects of Different Extracts of *Struthanthus quercicola*: A Preliminary Study

**DOI:** 10.1155/2022/9679739

**Published:** 2022-04-13

**Authors:** Luz Eugenia Alcántara-Quintana, Carely Arjona-Ruiz, Denisse de Loera, Rubí Gamboa-León, Yolanda Terán-Figueroa

**Affiliations:** ^1^CONACYT Chair Attached to Coordination for Innovation and Application of Science and Technology (Ciacyt), Autonomous University of San Luis Potosi, Av. Sierra Leona 550, Lomas de San Luis, CP 78210. San Luis Potosí, Mexico; ^**2**^ Photochemistry and Synthesis Laboratory, Faculty of Chemical Sciences, Autonomous University of San Luis Potosí, Av. Dr. Manuel Nava 6 Zona Universitaria, CP 78210, Mexico; ^3^Academic Coordination Southern Huasteca Region, Autonomous University of San Luis Potosi, Km. 5 Carretera Tamazunchale-San Martín, CP 79960. Tamazunchale, Mexico; ^4^Laboratory of Microbiology, Parasitology and Food Toxicology, Faculty of Nursing and Nutrition, Autonomous University of San Luis Potosi, Av. Niño Artillero 130, Zona Universitaria, CP 78240. San Luis Potosí, Mexico

## Abstract

*Struthanthus quercicola*, a hemiparasitic plant known as “seca palo,” is used by Nahuatl traditional healers against diabetes, wounds, and rashes. We aimed to investigate the effects of different *S. quercicola* extracts, which were selected based on their traditional use in Tamazunchale, San Luis Potosí, on the cell viability and antioxidant activity in HeLa cell cultures. *S. quercicola* growing on *Guazuma ulmifolia* and *Citrus* sp. hosts was collected, and methanolic and ethanolic extracts as well as decoctions, infusions, and microwave-assisted extracts were obtained. The terpenoid, alkaloid, flavonoid, saponin, and tannin contents of each extract were evaluated qualitatively and quantitatively. The effects of different extracts on the viability of cervical adenocarcinoma (HeLa) cells were tested using an MTT assay. The differences in the total flavonoid and phenolic contents and free-radical scavenging activity in relation to the host and the extract were also determined. In assessments of the effects of the extracts on cell viability, eight organic extracts (4 from *G. quercicola* grown on Host 1 and 4 from *G. quercicola* grown on Host 2) were shown to decrease cell viability significantly in comparison with the control. However, the extract obtained by percolation (PMeOH) caused a significant increase in cell viability (*p* < 0.05), especially with the plant grown on Host 1. The microwave aqueous and methanolic extracts of the plants grown on both hosts showed a significant increase in the percentage of apoptosis (*p* < 005). In conclusion, different extracts of *Struthanthus quercicola* showed variable effects on cell viability and apoptosis. Isolation of the molecule or molecules with inhibitory and proliferative effects on cells should be conducted to evaluate their possible use as antineoplastic agents.

## 1. Introduction

Humans have long used natural products, especially plants, for medical purposes and relied on these products to provide leads for drug discovery and development. A number of approved therapeutic agents show a natural product relationship since 21% of synthetic drugs are natural product mimics and 4% contain a pharmacophore from a natural product; additionally, 4% of approved drugs are unaltered natural products, 21% are derived from a natural product, and 1% are botanical drugs containing a defined mixture recognized as a drug entity by the U.S. FDA [1, 2].

Huasteca Potosina is a region of San Luis Potosí where traditional medicine is used to treat several illnesses. Alonso-Castro et al. identified 73 plant species used against several ailments such as diabetes, cough, epilepsy, arthritis, diarrhea, stomachache, vomiting, heart attack, body pain, and rash in Aquismon [3]. Some studies have also reported the antiproliferative, cytotoxic, and antitumor effects of Mexican plants, indicating their potential usefulness for cancer treatment [4–9].

*Struthanthus* Mart. species, also named mistletoe, are herbaceous plants that are scandent, dioecious, epiphytic, and foliated. The stems are cylindrical or quadrangular, and the inflorescences are spikes or racemes of three. The fruit is oval orange, red, or blue, and the seeds have bright green embryos and copious endosperms. These mistletoes are also known as “injertos” or “seca palo”. They are hemiparasitic plants (Loranthaceae) abundant in Mexico and could affect significant extensions of trees, reducing the production of cones and seeds and diminishing the diameter, height, and volume. The endophytic system invades the host vascular system and shoots regeneration from new endophyte buds [10]. This plant is native to Costa Rica, El Salvador, Guatemala, Honduras, Mexico Central, Mexico Northeast, Mexico Southeast, Mexico Southwest, Nicaragua, and Panama [11].

*Struthanthus quercicola*, known as seca palo, is used by Nahuatl traditional healers against diabetes, wounds, and rash. The administration is like infusion or decoction, which are prepared according to the recommendation of traditional healers (three 15 cm rods of the whole plant are boiled in 1 L of water for 20 min and are drunk throughout the day). This mistletoe (Loranthaceae) uses other plants as hosts and is classified as a hemiparasite. Traditional healers use plants growing on *Guazuma ulmifolia* and *Citrus* sp. Jiménez-Estrada et al. [12] reported the antiproliferative activity of *Struthanthus palmeri* on RAW 264.7 and L929 cell lines.

Considering its traditional use and that it has been reported to have an effect against cancer, the aim of this study was to investigate which type of antioxidants is present in different extracts of *S. quercicola* and the effect of these extracts on the cell viability in cervical cancer HeLa cell cultures.

## 2. Materials and Methods

### 2.1. Plant Material

The plants were selected based on their traditional use with the assistance of traditional healers (Martina Chaires from Aquismón, Teenek Region, and Santos Santiago Cruz and Eustolia Suviri from Tamazunchale, Nahuatl Region, both of which are regions in the state of San Luis Potosi, Mexico). Plants growing on *Guazuma ulmifolia* were collected in La Garita, Tambaque, Aquismón (−99.042778, 21.681111), and plants growing on *Citrus* sp. were collected in Enramaditas, Tamazunchale (−98.808056, 21.202500) in January 2016. The plants were taxonomically identified by the taxonomist José García Pérez at the Herbal “Isidro Palacios,” Desert Areas Research Institute, Autonomous University of San Luis Potosí, and a voucher of classification was assigned (Table 1).

### 2.2. Extract Preparation

The whole plant was dried at room temperature and protected from dust and sunlight. The dried material was ground using a manual mill (Estrella®). Five grams of each plant were macerated separately at room temperature using methanol and ethanol. Decoctions were obtained by boiling 5 g of plant material in 50 mL of distilled water for 20 min. Infusions were prepared by placing 1 g of the plant material in 50 mL of boiling water for 20 min. Microwave-assisted extraction was performed in a Mars 6 microwave reactor (CEM) using methanol and 5 g of the plant material with the following parameters: radiofrequency power, 350 W; temperature-time ramp, 10 min with a final temperature of 50°C (356 °F) held for 20 min. All extracts were filtered, the organic solvents were evaporated to dryness under reduced pressure, and the extracts were stored at −4°C in amber glass vials until analysis.  Table 2 lists the extract codes and yields.

### 2.3. Phytochemical Screening

#### 2.3.1. Test for Sterols and Terpenoids (Liebermann–Burchard test)

10 mg of the extract was dissolved in 2 mL of chloroform, after which 2 drops of acetic anhydride and concentrated sulfuric acid were carefully added. A reddish-brown color at the interface indicated the presence of sterols and terpenoids [13].

#### 2.3.2. Test for Alkaloids (Dragendorff Test)

Approximately 10 mg of the extract was warmed with 2% sulfuric acid for 2 min and then filtered. A few drops of Dragendorff's reagent were added, and an orange precipitate indicated the presence of alkaloids [14].

#### 2.3.3. Tests for Flavonoids

*Shinoda Test*. 10 mg of the extract was dissolved in 1 mL of methanol, a chip of magnesium metal was added, and a few drops of 0.5 N HCl were added. The presence of a pink magenta color indicated the presence of flavonoids [15].

*Constantinesco Test*. Three drops of 10% sodium acetate solution were added to 1 mL of the extract, followed by the addition of 3 drops of aluminum chloride 2.5%; the formation of a yellow color indicated the presence of flavonoids [16].

#### 2.3.4. Test for Saponins (Frothing Test)

Samples were mixed with 5 mL of water in a test tube, warmed, and shaken vigorously. The formation of a stable foam indicated the presence of saponins [17].

#### 2.3.5. Test for Tannins (Folin–Ciocalteu Test)

Five drops of Folin reagent and two drops of sodium carbonate 7.5% were added to the extract. The green color indicates the presence of phenols, light blue indicates moderate presence of phenols, and intense blue indicates the abundance of phenols [16].

### 2.4. Total Phenolic Content of the Extracts

The total phenolic content of the aqueous and organic extracts was determined by using the Folin–Ciocalteu reagent with the microplate method, as reported by Bobo-García et al. [18]. In this method, 100 *μ*L of the Folin–Ciocalteu reagent (1 : 4 diluted) was added to 20 *μ*L of the diluted plant extract (1 mg/mL) and shaken for 60 s in a 96-well microplate. The mixture was left for 240 s, after which 75 *μ*L of sodium carbonate solution (100 g/L) was added, and the mixture was shaken and left for 2 h at room temperature. The absorbance at 750 nm was measured using a spectrophotometer (Synergy H Y20003642T; BioTek). An ethanol solution was used as a blank. The phenolic content was calculated as gallic acid equivalent by comparison with a calibration curve of gallic acid standard solutions (10–100 *μ*g/mL) and was expressed as mg gallic acid equivalent per gram of dry extract. Samples were analyzed in triplicate. *Y* = 0.1072 + 0.1307, *R*^2^ = 0.9985.

### 2.5. Total Flavonoid Content of the Extracts

The aqueous extracts were used to determine the total flavonoid content using an aluminum chloride colorimetric assay adapted to a microplate method [16]. The plant extract (100 *μ*L, 1 mg/mL) was mixed with 100 *μ*L of AlCl_3_ (2%) in a 96-well microplate. The mixture was kept in a dark place at room temperature for 10 min. The absorbance at 365 nm was measured using a spectrophotometer (Synergy H Y20003642T; BioTek). An ethanol solution was used as a blank. The flavonoid content was calculated as quercetin equivalent by comparison with a calibration curve of quercetin standard solutions (5–45 *μ*g/mL) and was expressed as mg quercetin equivalent per gram of dry extract. Samples were analyzed in triplicate. *Y* = 0.0085x − 0.1554, *R*^2^ = 0.9965.

### 2.6. Free-Radical Scavenging Activity

Antioxidant activity was determined using the 2, 2-diphenyl-1-picryl-hydrazyl-hydrate (DPPH) microplate method as reported by Bobo-García et al. [18], with slight modifications. Diluted plant extract (100 *μ*L) was mixed with 100 *μ*L of DPPH solution (0.4 mM) and shaken for 60 s in a 96-well microplate. The mixture was kept in the dark at room temperature for 30 min. The absorbance at 517 nm was measured using a spectrophotometer (Synergy H Y20003642T; BioTek). An ethanol solution was used as a blank. DPPH free-radical scavenging activity was calculated as Trolox equivalent by comparison with a calibration curve of Trolox standard solutions (4–28 *μ*g/mL) and was expressed as mg Trolox equivalent per gram of dry extract. Samples were analyzed in triplicates. The DPPH radical scavenging assay was performed using a microplate method as reported by Bobo-García et al. [18], with slight modifications. Briefly, 100 *μ*L DPPH radical solution (0.4 mM) was mixed with 100 *μ*L of various concentrations of the extract sample dissolved in ethanol (4–30 *μ*g/mL). The mixture was kept in the dark at room temperature for 30 min. Absorbance was read at 517 nm using a spectrophotometer (Synergy H Y20003642T; BioTek). Ethanol and Trolox solutions were used as the blank and standard, respectively. The percentage inhibitory activity was calculated using equation (1), where *A*_0_ is the absorbance of the control, and  *A*_1_ is the absorbance of each sample extract. The half-maximal inhibitory concentration (IC_50_) was obtained by fitting a nonlinear regression using a four-parameter logistic function (equation (2)) and expressed in *µ*g/mL, where the parameter 10^(*x* − *LogC*)^ is the inflection point and the estimate of IC_50_.

(1) $$\begin{array}{c} \text{Inhibition \%}=A_{0}-\frac{A_{1}}{A_{0}}\times 100, \end{array}$$

(2) $$\begin{array}{c} Y=\frac{D+\left(A-D\right)}{1+10^{\left(x-LogC\right)B}}. \end{array}$$

### 2.7. Cell Line and Cell Culture

We used a HeLa cell line that was obtained several years ago for human cervical adenocarcinoma (CCL-2™; ATCC). HeLa cells were maintained in Dulbecco's modified Eagle's medium (DMEM D6429; Sigma-Aldrich) supplemented with 10% (v/v) fetal bovine serum (12103C; Merck-Millipore), 2 mM glutamine, 100 U/mL penicillin, and 100 *μ*g/mL streptomycin (P4083; Sigma-Aldrich) at 37°C, 5% CO_2_ in a humidified atmosphere. Cells (5 × 10^4^ cells/well) were cultivated in 96-well plates [19]. The cells were observed, and images were acquired (magnification, 20×) using an inverted microscope (Labomed TCM 400) and then separated by centrifugation at 600 × g for 6 min. The supernatant was gently removed, and the cell pellet was counted. Three replicates were used for each treatment.

### 2.8. Exposure

The cells were treated with extracts (100, 50, 25, and 12.5 *μ*g/mL) for 72 h.

### 2.9. Cell Viability Assay

After incubation for 72 h, 100 *μ*L (5.0 mg/mL) of 3-(4, 5-dimethylthiazol-2-yl)-2, 5-diphenyl tetrazolium bromide (MTT, 11465007001; Roche) was added to each well. After 4 h, the formazan product was dissolved in dimethyl sulfoxide, and the absorbance at 570 nm was measured [20] using a microplate reader (Multiskan; Thermo Scientific). Nontreated cells and cells treated with DMSO 0.01 *μ*g/mL (D9170; Sigma-Aldrich) were used as negative and positive controls, respectively. The assays were performed in triplicate in three independent experiments. The viability percentage was obtained by considering the control as 100%.

### 2.10. Apoptosis Detection Assays

Apoptotic cells were measured using an annexin V apoptosis kit according to the manufacturer's instructions (APOAF-20TST; Sigma-Aldrich). Briefly, the cells were washed once with PBS 1X and centrifuged at 13,000 rpm for 5 min. After that, they were washed once in 1X binding buffer and resuspended in 1X staining buffer. Then, 100 *µ*L (approximately 5 × 10^5^ cells) was incubated with 10 *µ*L of annexin V at room temperature for 15 min in the dark. The absorbance at 570 nm was measured using a microplate reader (Multiskan; Thermo Scientific). The assays were performed in triplicate in three independent experiments. The apoptosis percentage was obtained by using the control as 100%.

### 2.11. Statistical Analysis

For the analysis of variance, the ANOVA parametric test was used, and significance was assigned for a *p* value less than 0.05. GraphPad v.8.4 program was used.

## 3. Results and Discussion

Table 3 shows the results of qualitative phytochemical tests of the aqueous and organic extracts of *S. quercicola* harvested from two different hosts. The analysis showed the presence of alkaloids, flavonoids, and tannins. Saponins were present in the aqueous extracts, except in infusion and decoction from Host 1.

**Figure 1:**
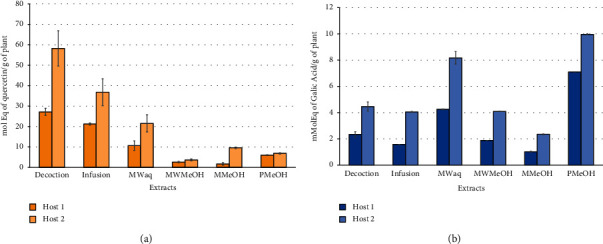
Total flavonoid (a) and phenolic (b) content of the aqueous and organic extracts of *S. quercicola* growing on *G. ulmifolia* (Host 1) and *Citrus* sp. (Host 2). Extract codes are defined in Table 2.

The data indicated differences in the total flavonoid content of the aqueous and organic extracts. Similar findings were obtained in comparison of the data for extracts from different hosts. As shown in Figure 1(a), the extracts from plants growing on *Citrus* sp. (Host 2) showed higher total flavonoid content than samples from plants growing on *G. ulmifolia* (Host 1). On the other hand, in Figure 1(b), in evaluations based on solvent polarity, since phenolic compounds were polar molecules, extracts prepared using polar solvents, such as water, methanol, and ethanol, were expected to have a higher phenolic content. Extracts from *S. quercicola* growing on Host 2 contained higher phenolic compound content than extracts from Host 1, regardless of the extraction method. For aqueous extracts, microwave extraction was the best extraction method for phenolic content, while percolation was the optimal method for organic extraction.

**Figure 2:**
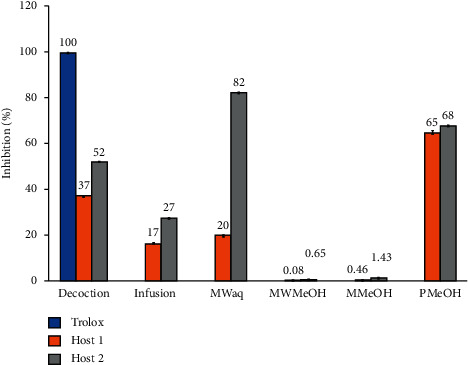
Antioxidant capacity with DPPH radical inhibition of *Struthanthus quercicola* extracts. Trolox served as the positive control.

The DPPH radical scavenging activity of the extracts is shown in Figure 2, and Table 4 shows the inhibition activity (IC_50_). The aqueous extracts showed higher activity than the organic extracts, while the extracts from *S. quercicola* grown on Host 2 exhibited greater antioxidant activity than those from plants grown on Host 1. The extract with the highest antioxidant activity was the aqueous extract from the plants grown on Host 2.

Extract codes are defined in Table 2.

**Figure 3:**
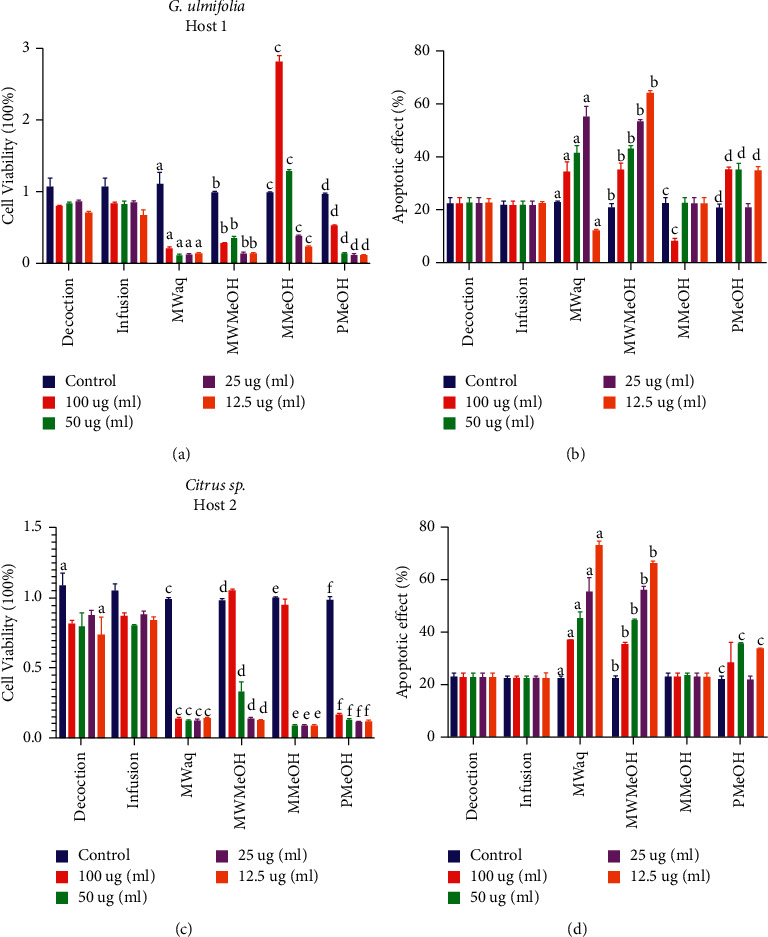
Effects of the extracts of *S. quercicola* growing on *G. ulmifolia* and *Citrus* sp. on cell viability and apoptosis. (a) and (c) HeLa cell viability. (b) and (d) Percentage apoptosis of HeLa cells. The cells were exposed to different concentrations of different extracts after 72 h of culture. The average value obtained in three experiments in triplicate is presented. Bars represent standard deviation. The letters above the bars indicate the extract and the concentration at which significance was observed. Extract codes are defined in Table 2.

The effects of the six extracts (two aqueous and four organic) from each plant were analyzed. In our analysis of the cell viability in HeLa cell cultures, the eight organic extracts (four each from *G. quercicola* grown on Host 1 and Host 2) caused a significant decrease in cell viability in comparison with the control. The maximum effect was observed with the six extracts from plants grown on Host 1; however, the organic extracts, specifically the percolation extract (PMeOH), caused a significant increase in cell viability (*p* < 0.05), especially the 100 *µ*g/mL extract from the plant grown on Host 1 (Figure 3(a) and 3(c)).

To corroborate these findings, tests were conducted to observe apoptosis in HeLa cell cultures grown in the presence of the different extracts. The annexin V binding assay is an indicator of the early stages of apoptosis. Translocation of phosphatidylserine (PS) from the inner face of the plasma membrane to the cell surface was determined. PS redistribution occurs earlier, and its externalization occurs during apoptosis induced by a variety of stimuli. The MWaq and MWMeOH extracts of the plants grown on both hosts showed a significant increase in the percentage of apoptosis (*p* < 005), reflecting the findings for cell viability, although the extracts from plants grown on Host 1 clearly caused higher percentages of apoptosis.

The 100 *µ*g/mL MMeOH extract of *S. quercicola* grown on Host 1 caused a decrease in apoptosis and an increase in cell viability (Figures 3(a) and 3(b)). In contrast, the PMeOH extracts of the plants grown on both hosts caused an increase in the percentage of apoptosis at 100, 50, and 12.5 *µ*g/mL (Figures 3(b) and 3(d)).

The documentation of traditional medicinal plants and remedies is becoming increasingly important because of the rapid loss of natural habitats [21]. Approximately 25% of the drugs prescribed worldwide are from plants [22]. Traditional medicine has a long history of use in Mexico. The document written by Bernardino de Sahagún “Historia de las cosas de la Nueva España” includes a description of the medicinal plants used by the Aztecs before the arrival of the Spaniards [23]. The different medicinal plants in Huasteca Potosina and their uses have been described previously [3]. Despite the current tendency to use plants with medicinal properties to cure different diseases, the safety of their use remains to be studied. In this regard, several properties have been attributed to the genus *Struthanthus* [24–26].

In relation to the findings of the present study, some of the extracts obtained from *S. quercicola* from two different hosts showed notable effects on the viability of HeLa cells, and the extracts that diminished cell viability did not have an apoptotic effect and vice versa, except for the 100 and 50 *µ*g/mL MMeOH extracts, which caused an increase in cell viability with no effect on the percentage of apoptosis.

The qualitative phytochemical studies carried out between the two decoctions showed that *S. quercicola* grown on Host 2 contains carbohydrates of sterols or triterpenes and greater amounts of flavonoids and tannins; in addition, the quantitative phytochemical analysis showed the same differences in the amount of flavonoids and total phenols, which explains why the antioxidant activity of the decoction of the plant grown on Host 2 was greater than that grown on Host 1.

Raffa et al. [27] have reported that flavonoids exhibit topoisomerase inhibitory activity and affect different signaling pathways such as AMP-activated protein kinase (AMPK), so the activity observed with the decoction of the plant grown on Host 2 could be attributed to the flavonoids present in it and the lack of activity of the decoction of the plant grown on Host 1 could be attributed to the absence of these compounds in its phytochemical composition through a saturable route. It is possible that the secondary metabolites that grant the inhibitory activity are different from those that grant antioxidant activity; these differences could also contribute to the differences in the antioxidant activity of extracts from plants grown on the two hosts. The same behavior could be seen among extracts obtained by maceration, with both extracts showing an inhibiting effect on viability, but the effect being significantly greater for the extract from the plant grown on Host 1. Based on the results obtained in the qualitative and quantitative analyses, the effects on cell viability could also be attributed to flavonoids.

The results of this study suggest that the effects of the extracts on cell viability and apoptosis in HeLa cells in culture are related to the extraction method since the microwave extracts showed reduced viability. Notably, these effects were not linked to the nature of the solvent (aqueous or organic) but were instead related to the polarity of these solvents since extracts obtained by methanolic percolation also had a negative effect on cell viability (these are separated by affinity to the solvent, so the metabolites extracted by methanol were polar, similar to those found in aqueous extractions). The antiproliferative activity of these extracts could be attributed not only to the flavonoids but also to the other polyphenols found in them since according to the quantitative analysis all of them contain polyphenols to some extent. Wang et al. [28] reported cytotoxic activity via apoptosis in HeLa cells by polyphenols; therefore, it is likely that the observed activity is attributed to these compounds.

Species of the genus *Struthanthus* exhibit various biological activities; for example, the hydroalcoholic extract of *S. vulgaris* contains flavonoids, tannins, and saponins with antimicrobial, antioxidant, and healing activities [29]. Another study reported the anti-inflammatory activity of extracts from this species but did not identify the secondary metabolites responsible for this activity [30]. The antioxidant and antiproliferative activities of the methanolic extract of *S. palmeri*, which contains flavonoids to which biological activity has been attributed, have also been investigated [12], and the hydroalcoholic extract of *S. venetus*, which is rich in polyphenols, has been reported to show antihypertensive [31] and antiproliferative activities [32].  Table 5 shows a summary of the classes of secondary metabolites identified as responsible for the biological activities reported in the species of the genus *Struthanthus*.

A previous study on the medicinal plants used in Huasteca Potosina, Mexico, described the species *Struthanthus densiflorus*. It has been traditionally used by people in Aquismón, San Luis Potosí, and Mexico for treating oral wounds, diabetes, and rash [3]. Traditional medicine includes the use of plants for the treatment and prevention of different types of cancer [33]. Plant-derived drugs of great importance, such as taxol isolated from *Taxus brevifolia Nutt.* (Taxaceae), have been used in the management of breast, ovarian, and brain cancers [34].

In Mexico, different extracts of *Cuphea aequipetala Cav.* (Lythraceae), known as the “cancer herb,” have been found to show slight cytotoxicity in a cervical cancer cell line and no cytotoxicity in nasopharyngeal carcinoma and colon cancer [35]. In an evaluation of five species of plants used in the state of Hidalgo, Mexico, to treat cancer, the crude ethanolic extract of the leaves of *Cupressus lusitanica* Klotzsch (Cupressaceae) showed cytotoxic effects in different cancer cell lines, and the cell death was shown to be related to apoptosis [36].

To date, the effects of a series of plant compounds at the molecular, cellular, or physiological level have not been evaluated, even though these compounds have shown potential in the treatment of conditions in humans [5]. In this study, we performed cellular-level assessments in culture and focused on cervical cancer. The effect of extracts of *S. quercicola* on HeLa cells has not been reported, nor have comparative studies been made from the same plant extracts obtained by microwave extraction and from two different hosts.

## 4. Conclusions

*S. quercicola* mainly contains metabolites such as alkaloids, flavonoids, and tannins. The concentrations of these metabolites vary depending on the host on which the plant grows, and, thus, the effects on cell viability and apoptosis also vary with the host plants. For aqueous extracts, microwave extraction was the best method for phenolic content, while percolation was the optimal method for organic compounds. Extracts from the plant growing on Host 2 contained higher phenolic compound content than extracts from Host 1; however, the maximum effect on HeLa cell viability was observed with the six extracts from plants grown on Host 1. Studies on the isolation of the molecule or molecules with inhibitory and proliferative effects on cells should be conducted to evaluate their possible use as antineoplastic agents in the treatment of cervical cancer.

## Figures and Tables

**Table 1:** Taxonomic classification of *Struthanthus quercicola*.

Kingdom	Plantae
Phylum	Magnoliophyta
Class	Magnoliopsida
Order	Santalales
Family	Loranthaceae
Genus	*Struthanthus*
Species	*Struthanthus quercicola* (Schltdl. & Cham.) Blume
Common name	Seca palo
Voucher	56,112 collected from *Citrus* sp.
	56,113 collected from *Guazuma ulmifolia*

**Table 2:** Extract yields of *S. quercicola* growing on *G. ulmifolia* and *Citrus* sp.

			*Guazuma ulmifolia*		*Citrus* sp.	
Code	Extract	Solvent	Host 1		Host 2	
			Weight (g)	Yield (%)	Weight (g)	Yield (%)
Decoction	Decoction	Water	0.2472	5	0.6763	13
Infusion	Infusion		0.8964	18	0.8092	16
MWaq	Microwave		0.6226	12	0.5637	11
MWMeOH		Methanol	0.7264	14	0.9736	19
MMeOH	Maceration		0.6092	12	1.3762	27
PMeOH	Percolation		100.00	15	33.00	16

MWaq, microwave water extract; MWMeOH, microwave water methanol extract; MMeOH, maceration methanol extract; PMeOH, percolation methanol extract.

**Table 3:** Qualitative phytochemical screening of extracts of *S. quercicola* growing on *G. ulmifolia* (Host 1) and *Citrus* sp. (Host 2).

Extract code^*∗*^	Sterols	Terpenoids	Alkaloids	Flavonoids	Saponins	Tannins
Host 1/Host 2						
Decoction	N/N	N/N	^*∗*^/^*∗*^	^*∗*^/^*∗*^	N/^*∗*^	^*∗*^/^*∗∗*^
Infusion	N/N	N/N	^*∗*^/^*∗*^	^*∗∗*^/^*∗∗∗*^	N/^*∗*^	^*∗∗*^/^*∗∗∗*^
MWaq	N/N	N/N	^*∗*^/^*∗*^	^*∗∗*^/^*∗∗∗*^	^*∗∗*^/^*∗∗∗*^	^*∗∗*^/^*∗∗∗*^
MWMeOH	^*∗∗*^/^*∗∗∗*^	^*∗∗*^/^*∗∗∗*^	N/^*∗∗*^	^*∗∗*^/^*∗∗∗*^	N/N	^*∗∗*^/^*∗∗*^
MMeOH	^*∗∗*^/^*∗∗*^	^*∗∗*^/^*∗∗*^	^*∗∗*^/^*∗∗*^	^*∗∗*^/^*∗∗*^	N/N	^*∗*^/^*∗∗*^
PMeOH	^*∗*^/^*∗*^	N/N	^*∗∗*^/^*∗∗*^	^*∗∗*^/^*∗∗∗*^	^*∗∗*^/^*∗∗*^	^*∗∗∗*^/^*∗∗∗*^

^*∗*^ = little coloration/precipitation, ^*∗∗*^ = intermediate coloration/precipitation, ^*∗∗∗*^ = abundant coloration/precipitation; N = no reaction/precipitation. Extract codes are defined in Table 2.

**Table 4:** *S. quercicola* extract free-radical scavenging activity.

Extract	IC_50_ (*µ*g/mL) ± SD	
	Host 1	Host 2
Decoction	363 ± 4	7 ± 0.89
Infusion	>500	69 ± 0.45
MWaq	124 ± 1.8	10 ± 2.4
MWMeOH	NA	NA
PMeOH	149 ± 0.31	55 ± 1.4
MMeOH	221 ± 5.2	149.5 ± 3.45
Trolox	14 ± 2	

**Table 5:** Classes of secondary metabolites identified as responsible for the biological activities reported in the species of the genus *Struthanthus*.

Species	Biological activities	Secondary metabolites	Study	Identified/Elucidate
*S. vulgaris* [20]	Antioxidant, antimicrobial, anti-inflammatory wound healing	Flavonoids, tannins, saponins	*In vitro*	Identified
*S. marginatus* [24]	Antiulcer	Flavonoids		
	Antituberculous			
*S. concinnus* [24]		Sterols, terpenoid triterpenes		Elucidate
*S. palmeri* [10]	Antioxidant	Flavonoids		Identified
	Antiproliferative			
S. venetus [22, 23]		Polyphenols	*In vivo*	
	Antihipertensive			
*S. vulgaris* [20]	Antioxidant	Flavonoids, tannins, saponins		Identified

## Data Availability

The data sets used and/or analyzed during the current study are available from the Faculty of Nursing of Autonomous University of San Luis Potosí, the research department, through the corresponding author on reasonable request.
